# Connections of the superior colliculus to shoulder muscles of the rat: a dual tracing study

**DOI:** 10.3389/fnana.2013.00017

**Published:** 2013-06-07

**Authors:** J. M. Rubelowski, M. Menge, C. Distler, M. Rothermel, K.-P. Hoffmann

**Affiliations:** ^1^Allgemeine Zoologie and Neurobiologie, Ruhr-University BochumBochum, Germany; ^2^Brain Institute and Department of Physiology, School of Medicine, University of UtahSalt Lake City, UT, USA; ^3^Tierphysiologie, Ruhr-University BochumBochum, Germany

**Keywords:** superior colliculus, m. rhomboideus, m. acromiodeltoideus, m. trapezius, pseudorabies virus, forelimb movements

## Abstract

Previous investigations indicate that the superior colliculus (SC) is involved in the initiation and execution of forelimb movements. In the present study we investigated the tectofugal, in particular the tecto-reticulo-spinal projections to the shoulder and arm muscles in the rat. We simultaneously retrogradely labeled the premotor neurons in the brainstem by injection of the pseudorabies virus PrV Bartha 614 into the m. rhomboideus minor and m. acromiodeltoideus, and anterogradely visualized the tectofugal projections by intracollicular injection of the tracer FITC dextrane. Our results demonstrate that the connection of the SC to the skeletal muscles of the forelimb is at least trisynaptic. This was confirmed by long survival times after virus injections into the muscles (98–101 h) after which numerous neurons in the deep layers of the SC were labeled. Transsynaptically retrogradely labeled brainstem neurons connected disynaptically to the injected muscles with adjacent tectal terminals were predominantly located in the gigantocellular nuclear complex of the reticular formation. In addition, putative relay neurons were found in the caudal part of the pontine reticular nucleus. Both tectal projections to the nucleus gigantocellularis and the pontine reticular nucleus were bilateral but ipsilaterally biased. We suggest this projection to be involved in more global functions in motivated behavior like general arousal allowing fast voluntary motor activity.

## Introduction

It is well known that the superior colliculus (SC) plays a key role in the visual grasp reflex involving movement of the eyes, the head, and the body (Akert, 1949; for a recent review see White and Munoz, 2011). More recently it has been shown that specific neurons in the intermediate and deep layers of SC are involved in reach movements of the arm (Werner, 1993; Werner et al., 1997a,b; Stuphorn et al., 2000). There are at least two potential pathways connecting the SC with the motoneurons in the spinal cord, the tectospinal tract and the tecto-reticulo-spinal tract.

The tectospinal tract is a very conserved structure present in all mammals investigated so far although it is better developed in predatory than in less predatory species (Waldron and Gwyn, 1969; Nudo and Masterton, 1988, 1989; Barton and Dean, 1993). It originates in the intermediate and deep layers mostly of the lateral SC and terminates almost exclusively on interneurons in the upper cervical cord whose motoneurons innervate neck muscles, and in the lower cervical enlargement which innervates the upper limb (Anderson et al., 1972; Rhoades and DellaCroce, 1980; Murray and Coulter, 1982; Nudo and Masterton, 1988; Rose et al., 1991; Xiulai et al., 1994; Muto et al., 1996; Yasui et al., 1998; Meredith et al., 2001). Another important tecto-spinal termination target are the propriospinal neurons in the C3-C4 segments of the cervical cord which govern target reaching of the forelimb (Illert et al., 1978; Alstermark et al., 2007; Alstermark and Isa, 2012).

The tecto-reticulo-spinal tract also originates in the intermediate and deep layers of the SC, involves the tegmental and pontine reticular formation and also ends in the upper cervical spinal cord (Nudo and Masterton, 1988; Keay et al., 1990; Redgrave et al., 1990; Yasui et al., 1998; Meredith et al., 2001) contacting at least in part the motoneurons of head extensor muscles (Anderson et al., 1971). In fact, the cells of origin of the tectospinal and the tecto-reticulo-spinal tract overlap to some degree in the intermediate and deep layers of the lateral SC (Yasui et al., 1998). Electrical stimulation of the SC and the dorsal tegmentum in the cat activates reticulospinal neurons (Schäfer, 1970; Udo and Mano, 1970) that in turn activate motoneurons of the flexor and extensor muscles of the hind- and forelimb (Grillner and Lund, 1968). Even though an at least disynaptic projection from the SC to the motoneurons of the fore- and hindlimb muscles was proposed based on electrical stimulation experiments (Anderson et al., 1972) the anatomical connection has yet to be demonstrated. This is the main aim of the present study.

During the last 20+ years viruses have been established as efficient transneuronal anterograde and retrograde tracers (Kuypers and Ugolini, 1990; Ugolini, 1995, 2010). In this study we used the attenuated pseudorabies virus PrV Bartha to transsynaptically label the chain of neurons connecting the SC with the shoulder and arm muscles. PrV Bartha is less virulent compared to wildtype PrV and a selective retrograde transsynaptic tracer, only propagating in the retrograde direction of information flow within the nervous system (Enquist, 2002). PrV Bartha has been extensively used in tracing studies of various neuronal circuits (e.g., Enquist, 1994, 2002; Marson and McKenna, 1996; Yang et al., 1999; DeFalco et al., 2001; Pickard et al., 2002; Cuthbertson et al., 2003; Damann et al., 2006; Kerman, 2008).

In our study we anterogradely labeled the first tectofugal synaptic sites using the tracer FITC dextrane, and premotor neurons of forelimb muscles by infection of proximal shoulder and arm muscles using PrV Bartha. We chose the m. trapezius, m. acromiodeltoideus, and m. rhomboideus because earlier experiments showed a high correlation of reach-related neuronal activity in the SC with the electromyographic activity of these muscles during a reach task in monkeys (Werner et al., 1997a; Stuphorn et al., 1999). With this approach we could demonstrate an at least trisynaptic signal transmission from the SC via relay neurons in the reticular nuclear complex and motor neurons in the spinal cord to the shoulder muscles.

## Materials and methods

All experiments were approved by the local authorities (Regierungspräsidium Arnsberg, local ethics committee) and were performed according to the European Communities Council Directive RL 2010/63/EC, the Deutsche Tierschutzgesetz of 7.26.2002, and the NIH guidelines for care and use of animals for experimental procedures.

### Animals

Altogether, 22 Wistar rats 5–12 weeks old and weighing 127–310 g were used. All animals had been bred and raised in the animal facility of the Department of Zoology and Neurobiology in an enriched environment.

In a first series of experiments (8 animals) we determined the time course of the retrograde propagation of the pseudorabies virus PrV-Bartha-614 in the tectofugal projection to the arm- and shoulder muscles. Subsequently, virus injections into the musculature were combined with FITC dextrane injections into the SC (14 animals) to visualize tectofugal projections.

### Recombinant virus

An attenuated recombinant strain of pseudorabies virus PrV Bartha expressing “monomeric red fluorescent protein 1” mRFP1 (Banfield et al., 2003) was used in the present study. The PrV-614 was propagated in monolayers of porcine kidney (PK15) cells, and assayed using a standard plaque assay. Viral stocks containing 8.3 × 10^8^ pfu/ml and 1.9 × 10^8^ pfu/ml [plaque forming units (pfu)] were aliquoted in 100 μl volumes and stored at −80°C. At the time of injection, viral aliquots were removed from the freezer and kept on ice until use.

### Surgery

The animals were premedicated with 0.05 mg atropine sulphate i.m. and initially anaesthetized with 90 mg/kg ketamine hydrochloride (Ketavet 10%, cp-pharma) and 5 mg/kg xylacine hydrochloride (Rompun®2%, Bayer). To ensure deep analgesia the animals received a bolus of 3 μg/kg fentanyl citrate (Fentanyl®, Jansen-Cilag). During the experiments, anaesthesia was maintained by boluses of 0.1 ml of 1% ketamine as needed. Body temperature was maintained at 36–37.5°, and heart rate was monitored during the whole experiment. Corneae were protected with ointment (VitA-POS®, Ursapharm).

### Tracer injections into the superior colliculus (SC)

After additional local anaesthesia with 0.1 ml bupivacain hydrochloride (Bupivacain® 0.5%, Jenapharm) the animals were placed in the stereotaxic apparatus, the skin overlying the skull was cut and a craniotomy was performed to allow access to the right SC. After localizing the SC electrophysiologically, the recording electrode was moved until a receptive field position in the lateral SC was reached (elevation 0 to −10°, azimuth 70–90°). In order to verify the location of tectospinal neurons, in 2 animals we electrically stimulated the right SC using low impedance recording electrodes or injection pipettes. Electrical stimuli consisted of single pulses (0.12 ms wide, 0.1–1.2 mA) or pulse trains (166 and 330 Hz, 0.04–0.2 mA, 200 ms duration). The electromyogram (EMG) of the left m. acromiotrapezius was recorded using silver wire electrodes.

Then the recording or stimulating electrode was replaced with a glass pipette connected to a Hamilton microsyringe, and 0.6–1.0 μl of dextrane (MW 3000) conjugated to fluorescein isothiocyanate (FITC) (15% in 0.1 m citrate-NaOH, pH 3.0) was injected at depths ranging from 400 to 2000 μm below the SC surface. A second injection was placed at 300–900 μm distance from the first injection. After completion of the injections the wound was closed in appropriate layers and protected with ointment containing neomycin sulphate (Nebacetin®, Yamanouchi Pharma). After full recovery, the animals were returned to their home cage.

### Virus injections into the shoulder muscles

**Figure 1:**
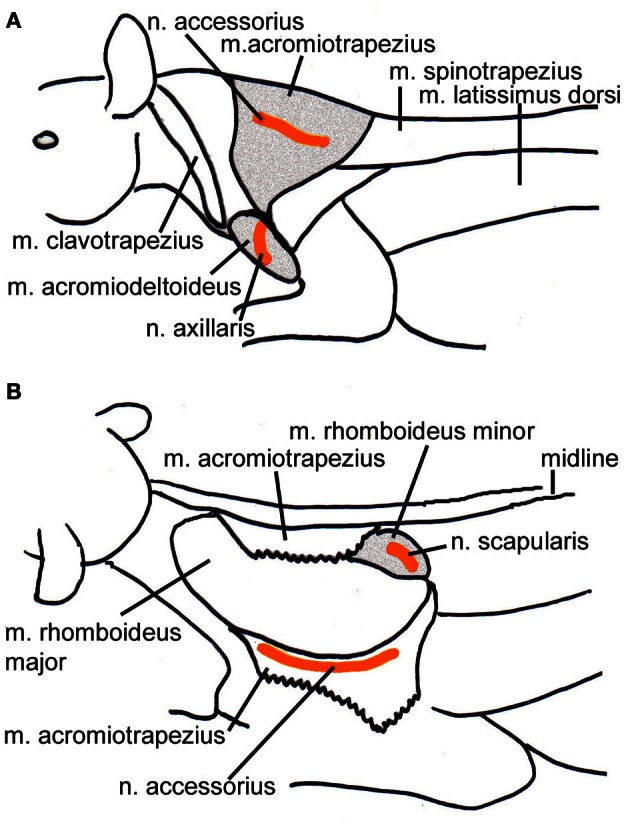
**Schematic drawings of the location of the injected muscles also indicating the position of the innervating nerves. (A)** Lateral view from the left depicting the m. acromiotrapezius and the m. acromiodeltoideus. **(B)** Dorsolateral view of the left side of the animal; the m. acromiotrapezius has been deflected to reveal the underlying m. rhomboideus minor. Relevant muscles are shaded, the course of the nerves is indicated in orange.

Forty-eight (to seventy-one) hours after the SC-injections the animals were re-anaesthetized as above. After additional local anaesthesia the skin overlying the left shoulder i.e., contralateral to the SC injections was cut and the m. trapezius, the m. acromiodeltoideus or the m. rhomboideus minor were exposed. In order to place the virus close to the innervation sites by the accessory nerve (m. trapezius), the axillary nerve (m. acromiodeltoideus), and the scapular nerve (m. rhomboideus) (Greene, 1935), respectively, injections were placed laterally in the m. trapezius, ventrally in the m. acromiodeltoideus, or medially and ventrally in the m. rhomboideus (Figure 1). Using a Hamilton microsyringe with a tapered tip 6 μl of the virus suspension PrV614 thawed immediately before the application were injected into the muscle. The needle was left in space for several minutes after the injection to avoid virus spill along the penetration track. Afterwards, the wound was closed in appropriate layers. After full recovery, the animals were returned to a cage in the biosafety level 2 lab and closely monitored during the entire survival time.

### Perfusion, histological processing and analysis

Eighty to one hundred hours (prior experiments) and 73–79 h (dual tracing experiments) after the muscle injections the animals were deeply anaesthetized with an overdose of halothane and transcardially perfused with 0.9% NaCl and 0.1% procaine hydrochloride, followed by 4% paraformaldehyde containing 57.6 g/l sucrose. The brain and the spinal cord up to C3 were removed, stored in 10, 20, and 30% sucrose, shock-frozen in isopentane, and stored at −70°C until use.

Three alternate series of 40 μm thick frontal sections were cut at a cryostat (Microm HM 500 OM) and used for fluorescence microscopy and cytoarchitecture. Sections were analyzed at a fluorescence microscope (ZEISS Axioskop) coupled to a computer based reconstruction system (Neurolucida, MicroBrightfields). Retrogradely labeled cells with neighboring labeled fibers and terminals were additionally scanned at a confocal microscope (LSM 510 Meta, ZEISS). Photomicrographs of labeled terminals (green) and neurons (red) were taken separately with a digital camera (AxioCam, ZEISS), and brightness and contrast were adjusted and noise suppressed with Photoshop (Adobe Vs. 5.5). Finally, the pictures were superimposed with ImageJ.

## Results

### Viral propagation

**Figure 2:**
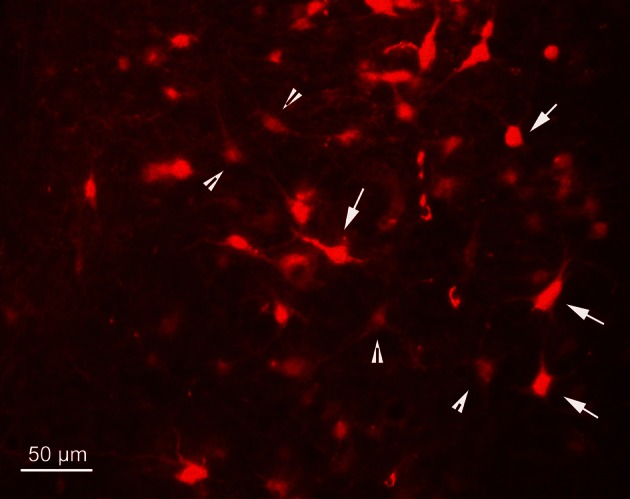
**Fluorescence photomicrograph demonstrating strongly labeled neurons (arrows) and less strongly labeled neurons (arrowheads) in the dorsal periaqueductal gray of rat SC2.** The neurons were transsynaptically labeled after PrV Bartha injection into the m. rhomboideus after a survival time of 100.5 h. Scale bar represents 50 μm.

**Figure 3:**
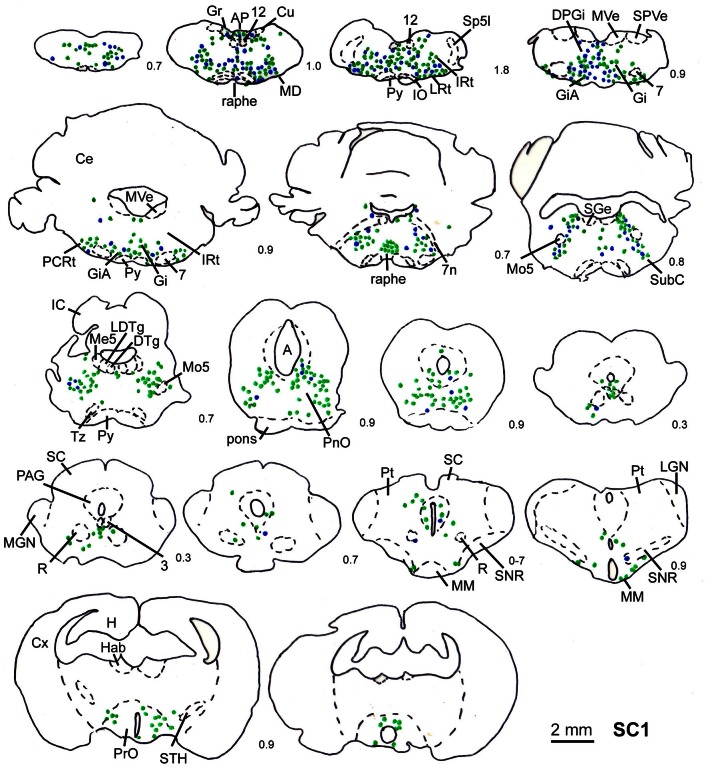
**Series of frontal sections through the brainstem and midbrain of rat SC1 demonstrating retrogradely labeled neurons after inoculation of the m. trapezius after a survival time of 80.3 h.** The sections are arranged from posterior (upper left) to anterior (lower right), the intersection distance in mm is indicated by the numbers between the sections. Blue symbols indicate strongly labeled neurons, green symbols indicate less strongly labeled cells. Scale bar indicates 2 mm. For abbreviations see list.

**Figure 4:**
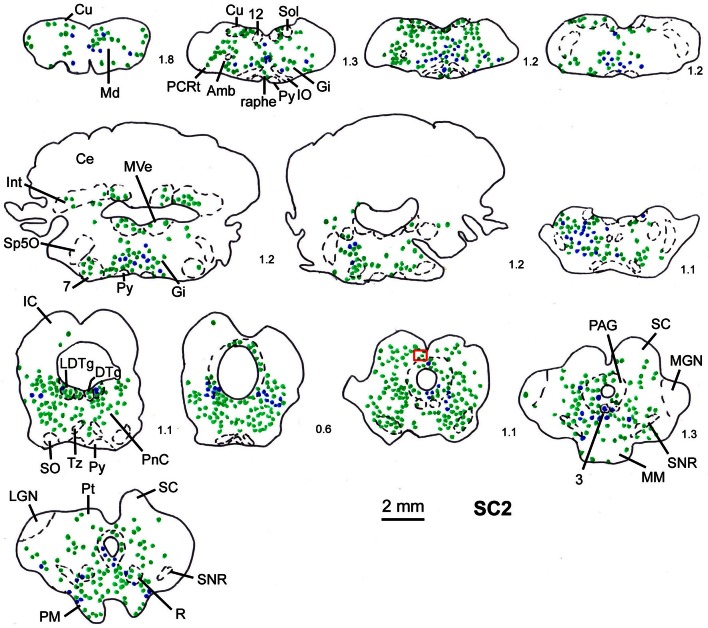
**Series of frontal sections through the brainstem and midbrain of rat SC2 demonstrating retrogradely labeled neurons after virus injection into the m. rhomboideus after a survival time of 100.5 h.** The red inset indicates the region where the photomicrograph depicted in Figure 1 was taken. Conventions as in Figure 3.

**Figure 5:**
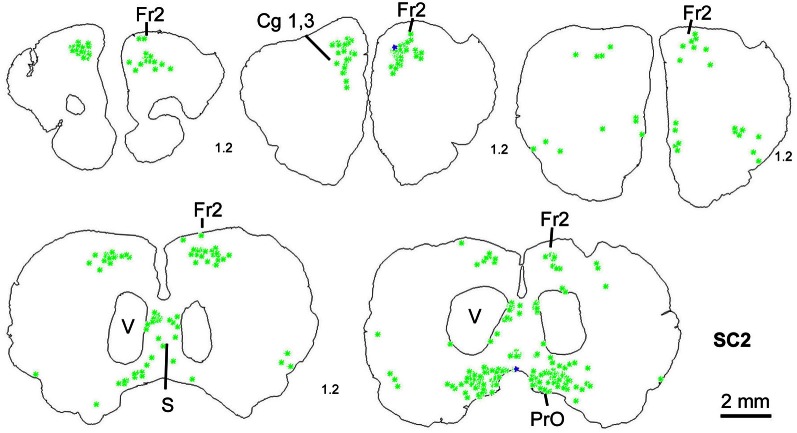
**Series of frontal sections through the frontal cortex of rat SC2 demonstrating retrogradely labeled neurons in frontal cortex after inoculation of the m. rhomboideus after a survival time of 100.5 h.** Conventions as in Figure 3.

In order to determine the velocity of the viral propagation, in a first set of experiments we varied the survival time of the animals after injection of the shoulder muscles (m. rhomboideus, m. trapezius). In 4 out of 8 animals the inoculation was successful, i.e., we detected retrogradely labeled cells in the brainstem, the SC and, in two cases, the motor cortex. Figure 2 shows examples of transsynaptically retrogradely labeled neurons in the midbrain of rat SC2 (see inset Figure 4). Based on the brightness of the fluorescent label we qualitatively distinguished between strongly labeled neurons (arrows in Figure 2) and weaker labeled cells (arrowheads in Figure 2). The extent of the labeling depended on the survival time. After 80–82 h, numerous labeled neurons were observed in the reticular formation, the raphe nucleus, and the periaqueductal gray. Sparse if any labeling was present in the SC and the preoptic area (SC1, Figure 3). In Figure 3 and the following figures blue symbols indicate strongly labeled neurons, green symbols represent qualitatively weaker labeled neurons. No label was observed in the motor cortex. After 98–101 h, a high density of labeled cells was observed in the reticular formation, the raphe nucleus, and the periaqueductal gray. Now, also the intermediate and deep layers of the SC as well as the preoptic area contained considerable numbers of retrogradely labeled cells (SC2, Figure 4). Labeled neurons were now also found in the frontal and cingulate cortex (Figure 5). These data indicate that, first, about 80 h after inoculation the first synapse of the tectofugal projection neurons had not been transgressed, and second that 16–18 h later a further retrograde level of the neuronal circuitry innervating skeletal muscles was labeled (i.e., the neurons projecting to the premotor neurons that project to the spinal interneurons or motoneurons).

### Orthodromic stimulation

In two cases (SC14, SC16) we used orthodromic electrical stimulation in the SC and recording of the EMG of the contralateral m. acromiodeltoideus to determine the location of tectospinal neurons. In SC14 we employed single pulses 0.15 ms wide at 0.1–1.2 mA. At a depth of 1500 μm below the SC surface the highest amplitude of the EMG was recorded with a latency of 5 ms. In SC16 we used pulse trains (166, 330 Hz; 100 μA; 200 ms duration). At a depth of 1000 μm below the SC surface, electrical stimulation caused movement of the whiskers. At a depth of 1250 μm movement of the contralateral front paw was observed. The strongest effect of electrical stimulation, i.e., spreading of the contralateral paw was elicited by stimulation at 2000 μm below the SC surface. These qualitative observations guided our approach for our anatomical experiments in which we made injections of FITC dextrane mainly into the deep layers of the SC to anterogradely label the projections into the brainstem. These locations yielded on average the highest frequency of varicosities in and close to nuclei containing the premotor neurons labeled after forelimb muscle injections.

### Dual tracer experiments

**Figure 6:**
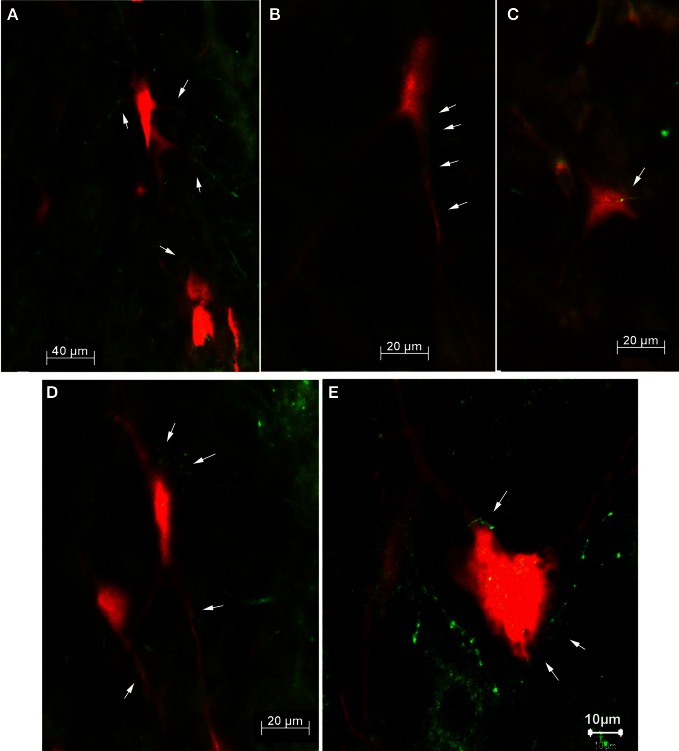
**Fluorescence photomicrographs (A–D) and confocal micrograph (E) of retrogradely labeled neurons (red) with adjacent tectofugal fibers and varicosities (green) from case SC14 (A,B,D,E) and from case SC16 (C).** The neurons were located in the n. reticularis gigantocellularis (Gi) **(B,D)**, in the n. reticularis gigantocellularis alpha (GiA) **(A,E)**, and in the n. reticularis intermedialis **(C)**, respectively. Arrows point to anterogradely labeled tectofugal fibers with varicosities.

**Figure 7:**
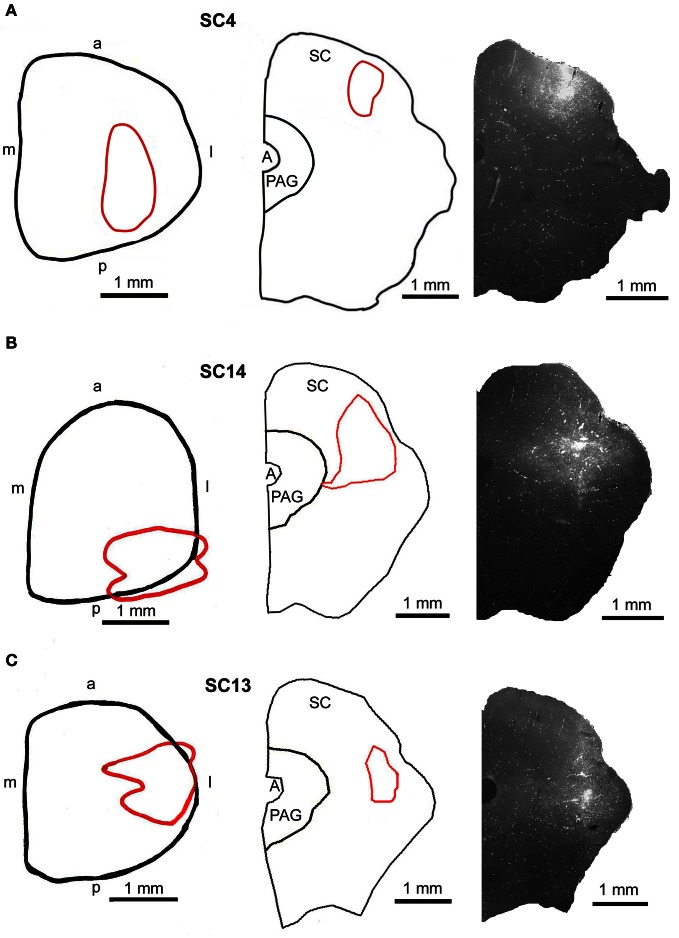
**Injection sites into the superficial (A, SC4), the intermediate-deep (B, SC14), and the deep (C, SC13) layers of the superior colliculus.** Left panel: reconstructions of the dorsal view of the right SC, middle panel: drawings of frontal sections through the center of the injection sites, right panel: fluorescent photomicrographs of the FITC injection sites. Red outlines in the left and middle row indicate the location of the FITC injections into the SC. a, anterior; p, posterior; l, lateral; m, medial; scale bars indicate 1 mm.

For the dual tracer experiments we chose survival times of 73–79 h after which no or only few retrogradely labeled neurons were observed in the SC in our initial experiments (see above) indicating that with these survival times the first tectofugal synapse was not overcome by the retrograde propagation of the virus. In this set of experiments we inoculated both the m. rhomboideus and/or the m. acromiodeltoideus (Table 1). Figure 6 demonstrates examples of premotor neurons retrogradely labeled by virus inoculation of m. rhomboideus and m. acromiodeltoideus. In close neighborhood of these neurons fibers and varicosities anterogradely labeled from the SC can be discerned. In the various cases FITC dextrane was injected at different depths in the SC (Figure 7; Table 1).

**Table 1:** **Summarized location of labeled neurons with neighboring varicosities ipsi- and contralateral to the SC injection site**.

**Area**	**Superficial-intermediate layers (cases SC3, SC4, SC8)**		**Intermediate–deep layers (cases SC10, SC11, SC14, SC16)**		**Deep layers (cases SC13, SC15)**	
	**ipsi**	**contra**	**ipsi**	**contra**	**ipsi**	**contra**
Gi	3	6	9	4	6	1
GiA	–	–	15	8	3	3
GiV	2	–	5	–	6	4
IRt	1	3	7	–	–	–
LRt	–	–	2	–	2	–
PCRtA	–	–	–	1	–	–
MdD	–	1	–	–	1	–
MdV	1	–	3	1	1	1
PMn	–	–	2	–	2	5
PnC	–	–	11	5	4	3
PnO	–	–	3	–	–	3
PnV	–	–	4	2	1	–
LPGi	–	1	2	3	–	–
DPGi	–	1	–	–	–	–
N. raphe	1		12		7	
DMTg	–	–	1	1	–	–
PAG	2	–	–	–	–	–
Mo5	–	–	1	–	–	–
SubC	–	–	–	2	3	–
other	1	–	3	4	6	2

#### Injections into the m. rhomboideus or the m. acromiodeltoideus and the superficial and intermediate layers of the SC.

**Figure 8:**
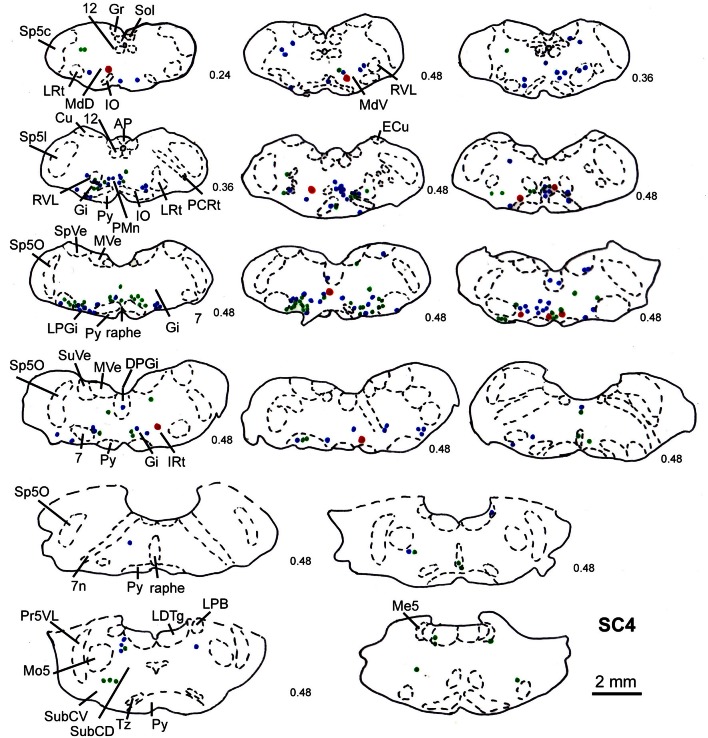
**Series of frontal sections through the brainstem and midbrain of case SC4 demarcating the location of retrogradely labeled neurons after inoculation of the left m. acromiodeltoideus (survival time of 79 h after inoculation) and FITC labeled varicosities after injection into the superficial layers of the right SC (2 days before the muscle inoculation).** Red symbols indicate retrogradely labeled cells with adjacent tectofugal fibers and varicosities. Other conventions as in Figure 3.

Predominantly the superficial layers of SC were injected in 2 cases (SC3, SC4) and the superficial and intermediate layers in case SC8 (Table 1). Even though the muscles injected in these cases differed the resulting labeling was comparable. Therefore, we here only present the data of case SC4. The injection site is depicted in Figure 7A in a reconstruction of the dorsal view of the SC (left), in a section through the center of the injection (middle), as well as in a photomicrograph (right). Red outlines indicate the FITC injection site. The resulting labeling is demonstrated in the frontal sections shown in Figure 8. The majority of cells labeled by muscle inoculation were located in the nuclei of the reticular formation, i.e., in the ventral medullary reticular nucleus (MdV), the paramedian reticular nucleus (PMn), and the gigantocellular reticular nucleus (Gi). Fewer neurons were labeled in the lateral (LRt) and the intermedial reticular nucleus (IRt), the lateral paragigantocellular nucleus (LPGi), and in the raphe nucleus. Sparse labeling was found in the dorsal medullary (MdD) and the parvocellular reticular nucleus (PCRt), the dorsomedial tegmental area (DMTg), the vestibular nuclei, and the central pontine gray. Altogether, 11 neurons with adjacent fibers and varicosities labeled from the SC were found in this case, most of which were located in Gi (*n* = 7), as well as in IRt (*n* = 1), MdD (*n* = 1), MdV (*n* = 1), and DPGi (*n* = 1).

The location of labeled neurons with neighboring varicosities found in cases SC3, SC4, and SC8 is summarized in Table 1.

#### Injections into the m. acromiodeltoideus or both m. acromiodeltoideus and m. rhomboideus and the intermediate and deep layers of the SC

**Figure 9:**
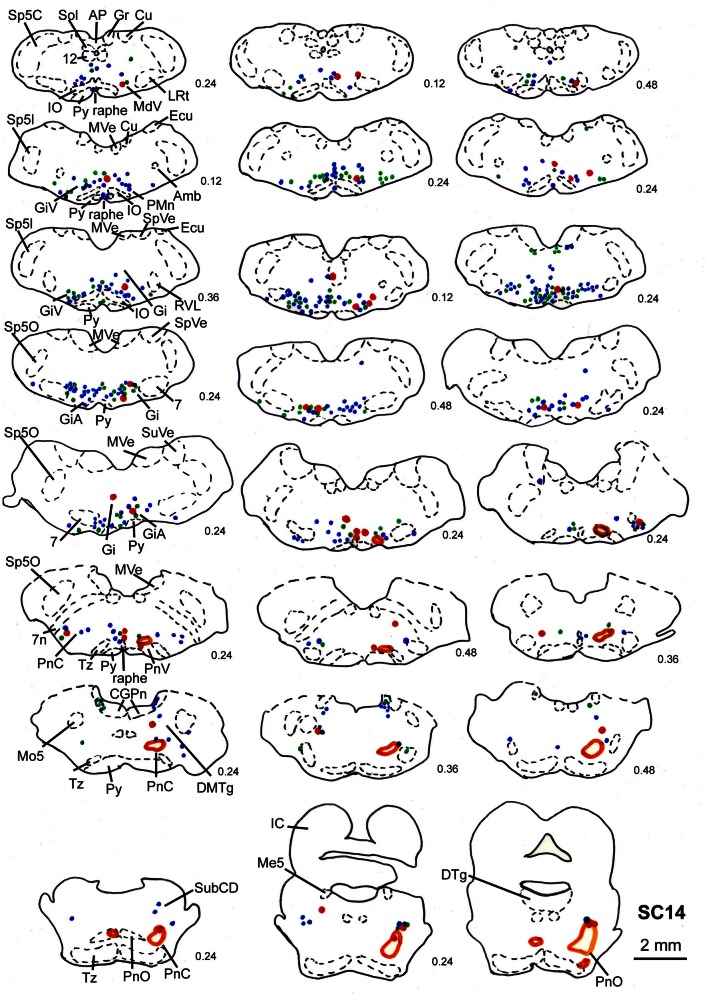
**Series of frontal sections through the brainstem and midbrain of case SC14 showing retrogradely labeled neurons after virus injection into the left m. acromiodeltoideus and the left m. rhomboideus, and FITC injection into the intermediate and deep layers of the right SC after a survival time of 73 h after inoculation.** Orange outlines indicate dense tectofugal terminal fields. Conventions as in Figures 3, 8.

In 4 more cases (SC10, SC11, SC14, SC16) the tracer injections encompassed the intermediate and deep layers of the SC. In case SC10 and SC11, retrograde labeling from the acromiodeltoid was rather sparse with most cells again being found in Gi, the ventral and the alpha subnucleus of Gi (GiV, GiA), and the LRt. A total of 15 neurons with neighboring varicosities were present in the reticular formation (Gi, GiA, GiV, IRt, LRt) and the DMTg. Significantly denser labeling was achieved by simultaneous inoculation of the m. rhomboideus and m. acromiodeltoideus in case SC14 and SC16. The tracer injection into the SC of case SC14 is demonstrated in Figure 7B, the resulting labeling is depicted in the frontal sections shown in Figure 9. Case SC14 yielded the strongest labeling of our experimental series, the distribution of the label, however, strongly resembled the other cases. Retrogradely labeled neurons were found mainly in the Gi, GiA, and GiV, with a few scattered neurons outside the reticular formation. The intracollicular tracer injection anterogradely labeled terminal fields in the ipsilateral GiA and the ipsilateral PnC/PnO as well as a smaller terminal field in the contralateral PnO. Altogether, 65 neurons with neighboring varicosities were found, 51 of which were located in the reticular formation. Most of these neurons lie in the GiA, the PnC, the PnO, Gi, and MdV. In addition, neurons with varicosities were found in the n. raphe, isolated neurons lay in the facial and the trigeminal nucleus, the DMTg, and the n. subcoeruleus. The SC injection in case SC16 confirmed the results from SC14. The data of the injections into the intermediate-deep collicular layers are summarized in Table 1.

#### Injections into both the m. acromiodeltoideus and the m. rhomboideus and into the deep layers of the SC

**Figure 10:**
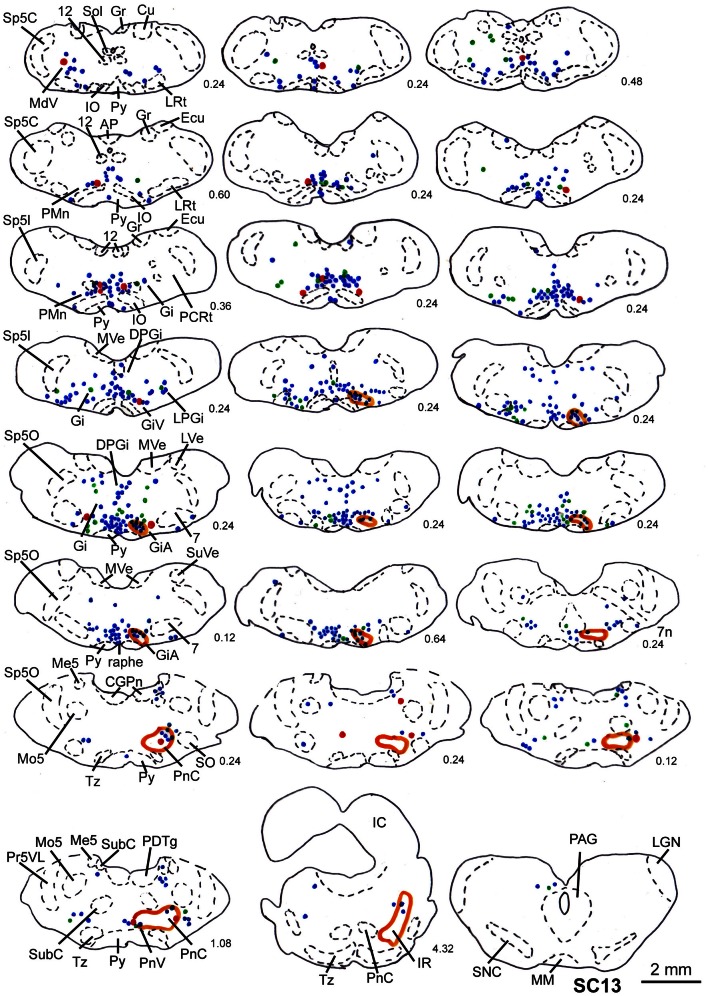
**Series of frontal sections through the brainstem and midbrain of case SC13 showing retrogradely labeled neurons after virus injection into the left m. acromiodeltoideus and left m. rhomboideus (survival time of 73 h after inoculation), and FITC labeled varicosities after injection into the deep layers of the right SC.** Orange outlines indicate dense tectofugal terminal fields. Conventions as in Figures 3, 8.

In two animals (SC13, SC15), the tracer injections were restricted to the deep layers of the SC. These injections were combined with inoculation of both the m. rhomboideus and the m. acromiodeltoideus. Even though the resulting amount of labeled neurons was much higher in SC13 than in SC15, the distribution of the cells was very similar. We therefore demonstrate only case SC13 (Figures 7C, 10). Again, the majority of labeled neurons lay in Gi, GiA, GiV, and the raphe nucleus, with additional cells in DPGi, PnV, and PnD. The SC injection resulted in labeling of distinct terminal fields in the ipsilateral GiA and PnC/PnV. A total of 32 retrogradely labeled neurons with neighboring varicosities were identified, 26 of which were located in the reticular formation, mainly in GiV, PMn, PnC, followed by Gi, GiV, MdV, PnV, the raphe nucleus, and the n. subcoeruleus. The results of cases SC13 and SC15 are summarized in Table 1.

Comparing all cases, two observations become apparent. First, the neurons projecting to the skeletomuscular system of the forelimb and potentially receiving input from the SC are mainly located in the various nuclei of the reticular formation and the raphe nucleus. Their distribution is ipsilaterally biased (104 ipsi- vs. 59 contralateral; *p* < 0.02 in a χ^2^-test). Second, SC injections into the deep layers on average yield stronger brain stem projections than injections into the intermediate/deep or the superficial/intermediate layers.

## Discussion

There are at least three disynaptic pathways from the deep layers of the SC to the motoneurons innervating the muscles of the shoulder and forelimb: first, the tectospinal projection via interneurons in the cervical enlargement; second, the tectospinal projection via propriospinal neurons in segments C3-C4, and third, the tecto-reticulo-spinal projection. In the present study we directly demonstrate for the first time the relay stations of the tecto-reticulo-spinal pathway to the skeletomotor system in the rat by simultaneously labeling tectal efferents with an anterogradely transported tracer, and the premotor neurons to the shoulder and arm muscles by inoculation with the transsynaptic retrograde tracer PrV Bartha. Retrogradely labeled neurons with adjacent tectofugal fibers and varicosities, i.e., presumed terminals were predominantly located in the reticular formation, mainly in the gigantocellular nuclear complex, and the raphe nucleus indicating an at least trisynaptic innervation of the shoulder muscles.

### Methodological considerations

In our experiments we used the attenuated alpha-herpes virus PrV Bartha that has been used in various systems including the locomotor system (Jovanovic et al., 2010). Pseudorabies virus supposedly has several disadvantages compared to rabies virus including infection of glia and epithelial cells, sensory and motor neurons, neuronal degeneration, and local spread (Ugolini, 2010). We chose an attenuated virus stock with high titer (10^8^ pfu/ml) to maximize the transneuronal transfer, and placed the inoculation close to or in the nerves supplying the various muscles therefore minimizing unspecific spread. To really limit the retrograde viral propagation to two synapses the strategy of genomic deletion of the gene encoding a glycoprotein (Gly) essential for transsynaptic spread should have been applied (Callaway, 2008; Arber, 2012). Degenerating virus-labeled cells were mainly found in our prior experiments after long survival times (>100 h) but were much less numerous with survival times around 80 h (data not shown). In our dual tracing experiments labeled tectofugal terminals were found adjacent to putatively intact neurons. The resulting retrograde labeling in the brainstem differed quantitatively but was qualitatively very similar in all cases thereby justifying the use of PrV Bartha in our tracing system. The reported amount of labeled cells with adjacent tectofugal varicosities probably is an underestimate because of two reasons. First, using an mRFP1 labeled virus strain in combination with an anterograde tracer coupled to the fluorescent dye FITC allowed us to directly view the resulting label. However, fading of the fluorescence limited viewing time. Thus, mainly strongly labeled cells and varicosities were detected. Second, the retrograde label was usually limited to the soma and proximal dendrites of the cells. As most axon terminals contact the dendrites of their target cells, these contacts remained undetected. Nevertheless, the constancy of the label across cases indicates that our results are highly reproducible.

### Comparison with other studies

Our dual tracing results are in accordance with electrophysiological studies. Electrical stimulation of the SC activates spinal motoneurons and thus influences movements of the limbs (e.g., Anderson et al., 1972; Sinnamon, 1984; Courjon et al., 2004; Philipp and Hoffmann, unpublished observation) and the neck (e.g., Anderson et al., 1971). This influence seems to be mediated by the reticular formation that receives input from the SC (Peterson et al., 1971) and in turn projects to the motoneurons supplying muscles of the neck, back, fore- and hindlimbs (Peterson et al., 1979). Especially neurons in the n. reticularis pontis caudalis and in the dorsorostral part of the gigantocellular reticular nucleus monosynaptically innervate motoneurons of the axial and limb musculature. Neurons in the pontomedullary reticular formation are modulated during limb movements (Buford and Davidson, 2004; Schepens and Drew, 2004; Schepens et al., 2008), and micro-stimulation in this region elicits muscle activity in the limbs as well as head movements and influences gaze shifts (Drew and Rossignol, 1990a,b; Freedman and Quessy, 2004; Quessy and Freedman, 2004; Davidson and Buford, 2006). A tecto-reticulo-spinal pathway for head orienting movements was also described anatomically (Perkins et al., 2009). A recent study in mouse implicates the precuneiform nucleus in the mesencephalic locomotor region in the generation of limb movements (Liang et al., 2012). However, in none of our dual tracer cases did we find neurons retrogradely labeled from the shoulder muscles with adjacent tectal projections in this region indicating that the precuneiform nucleus is not part of the shortest tectofugal pathway supplying shoulder muscles.

Our successful SC-injection sites were located in the lateral part of the SC where in primates reach-related activity can be recorded in the intermediate and deep layers (Werner, 1993; Werner et al., 1997a,b) and where microstimulation elicits arm movements (Philipp and Hoffmann, unpublished observation). These injections labeled circumscribed fields with dense terminals mainly if not exclusively ipsilaterally in the GiA and the PnC confirming data by Kawamura et al. (1974) and Redgrave et al. (1990). Relatively few neurons retrogradely labeled from the forelimb muscles were detected within these terminal fields, most neurons with adjacent terminals were found in other regions of the GiA and PnC which only showed a moderate ipsilateral bias.

### Tecto-spinal or tecto-reticulo-spinal connection?

Transneuronal labeling with viruses allows visualization of not only first-order, but also second- and third-order neurons with comparable labeling strength because viruses replicate after infecting new neurons after crossing synapses (Ugolini, 1995). This property also indicates that the velocity of the transneuronal propagation in a system depends on the number of synapses crossed. In our first series of experiments we varied survival time between 80 and 100 h after inoculation. With shorter survival times (80 h) retrogradely labeled neurons were mainly present in the reticular formation and in the raphe nucleus but neither in SC nor in the cerebral cortex. After longer survival labeled neurons were also present in the SC and the frontal and cingulate cortex indicating that at least one additional synapse had been crossed. In a recent study in neonatal mice the time course of transsynaptic propagation of PrV Bartha after inoculation of hindlimb muscles was investigated in detail (Jovanovic et al., 2010). Motoneurons in the lumbar spinal cord were labeled after 24–32 h, interneurons projecting monosynaptically to the motoneurons were encountered after 36–40 h indicating that the transsynaptic propagation over one synapse takes about 12 h. Infection and propagation time varies with age and species (Ugolini, 1995). In young adult rats, premotoneuronal cells were labeled after 52–72 h after inoculation of various muscles and the sciatic nerve (e.g., Dobbins and Feldman, 1994, 1995; Kim et al., 2000; Chamberlin et al., 2007). This indicates that with our survival times of 72–82 h the labeled neurons in the reticular formation are indeed premotoneurons, also considering the fact that viral replication may vary between injections into the periphery (i.e., the muscles) and into the nervous system.

Our data confirm earlier data that in rat direct cortico-motoneuronal connections are not established (e.g., Yang and Lemon, 2003; Alstermark et al., 2004). With survival times of 72–79 h we did not find retrogradely labeled neurons in the SC. This clearly demonstrates that the tecto-motoneuronal projection in rat is subtle at best. In contrast, our experiments clearly support a robust tecto-spinal and tecto-reticulo-spinal pathway with at least 3 synapses between SC and forelimb muscles. In the tecto-reticulo-spinal pathway most retrogradely labeled neurons with adjacent FITC labeled varicosities that presumably represent input from the SC were located in the gigantocellular complex of the reticular formation as well as in PnC.

### Functional considerations

In rodents, there appears to exist a functional segregation of the SC, with the medial SC and its representation of the dorsal visual field being responsible for detection of putative predators, and the lateral SC representing the horizontal and ventral visual field being assigned to the detection of possible prey and social partners (Dean et al., 1989). In line with this hypothesis, Comoli and coworkers could demonstrate in a recent study (Comoli et al., 2012) that the defense region in the medial SC and the approach-related region in the lateral SC receive distinct cortical and subcortical afferents with very little overlap between the two regions. Using electrical stimulation, Sahibzada and colleagues found that contralateral orienting and approach behavior could be elicited mainly from the intermediate and deep layers of the rostral and central SC and from all layers but the deep white in caudal SC. By contrast, mainly ipsilateral avoidance and defense behavior was seen after stimulation of the superficial and intermediate layers of the rostral SC, the intermediate and deep layers of central SC, and the deep layers of the caudal SC (Sahibzada et al., 1986). Comparing their results with anatomical and lesion evidence the authors suggest that orientation and approach movements are mediated via the crossed tectofugal pathway whereas avoidance involves the ipsilateral tectofugal projections. In this respect, rodents may well differ from e.g., primates or carnivores due to the quite different predator-prey ecology.

Our present experiments show that premotoneuronal neurons in the reticular formation transsynaptically labeled from the shoulder muscles receiving tectofugal afferents are more often found ipsilaterally to the injected SC than contralaterally. This could imply that this pathway is more involved in avoidance than in appetitive behavior indicated by reaching. Nevertheless, our SC injections were always placed in the lateral SC with receptive fields below the horizon that are suited to register food or conspecifics. Interestingly, most neurons connecting the SC to motoneurons of forelimb muscles were found in the nucleus gigantocellularis. In a recent paper, Pfaff et al. (2012) describe the importance of neurons in this nucleus which may have originated from the Mauthner cells in fish for general arousal in mammals. Our data do not allow us to decide about the exact function of the projections revealed in the present study but the bilateral, (though ipsilaterally biased) projection suggests a more global function in motivated behavior like preparing for action or sensory-guided locomotor decisions (Felsen and Mainen, 2008). Further experiments with electrical stimulation or, better still, applying optogenetic methods to lateral vs. medial regions of the SC in behaving animals could help to elucidate this matter.

### Conflict of interest statement

The authors declare that the research was conducted in the absence of any commercial or financial relationships that could be construed as a potential conflict of interest.

## Abbreviations

3: nucleus oculomotorius
7: nucleus facialis
7n: nervus facialis
12: nucleus hypoglossus
A: aqueduct
Amb: nucleus ambiens
AP: area postrema
Ce: cerebellum
Cg1,3: cingulate cortex, area 1, 3
CGPn: pontine central gray
Cu: nucleus cuneatus
Cx: cortex
DMTg: dorsomedial tegmental area
DPGi: nucleus paragigantocellularis pars dorsalis
DR: dorsal raphe nucleus
DTg: nucleus tegmentalis dorsalis
Ecu: nucleus cuneatus externus
Fr2: frontal cortex, area 2
Gi: nucleus gigantocellularis pars reticularis
GiA: nucleus gigantocellularis pars reticularis alpha
GiV: nucleus gigantocellularis pars reticularis pars ventralis
Gr: nucleus gracilis
H: hippocampus
Hab: habenulae
IC: inferior colliculus
Int: nucleus interpositus
IO: oliva inferior
IRt: nucleus reticularis intermedialis
LDTg: nucleus tegmentalis laterodorsalis
LGN: nucleus geniculatus lateralis
LPB: nucleus parabrachialis lateralis
LPGi: nucleus paragigantocellularis pars lateralis
LRt: nucleus reticularis pars lateralis
LVe: nucleus vestibularis lateralis
Md: nucleus reticularis medullaris
MdD: nucleus reticularis medullaris pars dorsalis
MdV: nucleus reticularis medullaris pars ventralis
MGN: nucleus geniculatus medialis
MM: mammillary body
Me5: nucleus trigeminus mesencephalicus
Mo5: nucleus trigeminus motorius
MVe: nucleus vestibularis medialis
PAG: periaqueductal gray
PCRt: nucleus reticularis parvocellularis
PDTg: nucleus tegmentalis posterodorsalis
PM: premammillary body
PMn: paramedian reticular nucleus
PnC: nucleus reticularis pontis pars caudalis
PnO: nucleus reticularis pontis pars oralis
PnV: nucleus reticularis pontis pars ventralis
pons: nuclei pontis
Pr5VL: nucleus trigeminus principalis ventrolateralis
PrO: area praeoptica
Pt: pretectum
Py: pyramidal tract
R: nucleus ruber
raphe: raphe nuclei
RVL: nucleus reticularis rostroventrolateralis
S: septum
SC: superior colliculus
SGe: nucleus supragenualis
SNC: substantia nigra pars compacta
SNR: substantia nigra pars reticularis
SO: oliva superior
Sol: nucleus tractus solitarii
Sp5C: nucleus trigeminus spinalis pars caudalis
Sp5I: nucleus trigeminus spinalis pars interpolaris
Sp5O: nucleus trigeminus spinalis pars oralis
SPVe: nucleus vestibularis spinalis
STH: nucleus subthalamicus
SubC: nucleus subcoeruleus
SubCA: nucleus subcoeruleus pars anterior
SubCD: nucleus subcoeruleus pars dorsalis
SubCV: nucleus subcoeruleus pars ventralis
SuVe: nucleus vestibularis superior
Tz: trapezoid body.
